# Rehabilitation and primary care treatment guidelines, South Africa

**DOI:** 10.2471/BLT.22.288337

**Published:** 2022-08-22

**Authors:** Thandi Conradie, Maria Charumbira, Maryke Bezuidenhout, Trudy Leong, Quinette Louw

**Affiliations:** a Division of Physiotherapy, Department of Health and Rehabilitation Sciences, Faculty of Medicine and Health Sciences, Stellenbosch University, Francie van Zijl Drive, Tygerberg, Cape Town, 7505, South Africa.; b Rural Rehab South Africa, Manguzi, South Africa.; c Essential Drugs Programme, South African National Department of Health, Pretoria, South Africa.

## Abstract

The World Health Organization recognizes rehabilitation as an essential component of universal health coverage (UHC). In many countries, UHC builds on a standard benefits package of services that is informed by the country’s essential medicines list, standard treatment guidelines and primary health care essential laboratory list. In South Africa, primary health care is largely provided and managed by primary health-care nurses and medical officers in accordance with primary health care standard treatment guidelines. However, rehabilitation is mostly excluded from these guidelines. This paper describes the 10-year process that led to rehabilitation referral recommendations being considered for inclusion in South Africa’s primary health care standard treatment guidelines. There were five key events: (i) a breakthrough moment; (ii) producing a scientific evidence synthesis and formulating recommendations; (iii) presenting recommendations to the national essential medicines list committee; (iv) mapping rehabilitation recommendations onto relevant treatment guideline sections; and (v) submitting revised recommendations to the committee for final consideration. The main lesson learnt is that, by working together, rehabilitation professionals can be of sufficient number to make a difference, improve service delivery and increase referrals to rehabilitation from primary health care. A remaining challenge is the lack of a rehabilitation representative on the national essential medicines list committee, which could hamper understanding of rehabilitation and of the complexities of the supporting evidence.

## Résumé

L'Organisation mondiale de la Santé considère la réadaptation comme un élément indispensable de la couverture sanitaire universelle (CSU). Dans de nombreux pays, la CSU repose sur un modèle de prestations de services qui tient compte de la liste nationale de médicaments essentiels, des directives thérapeutiques normalisées et d'une liste des analyses en laboratoire essentielles dans le cadre des soins primaires. En Afrique du Sud, les soins primaires sont majoritairement assurés et gérés par du personnel infirmier et médical, conformément aux directives thérapeutiques normalisées en la matière. En revanche, la réadaptation n'y figure généralement pas. Le présent document décrit les dix années de processus qui ont permis d'émettre des recommandations de référence relatives à la réadaptation, que l'Afrique du Sud envisage d'inscrire dans ses directives thérapeutiques normalisées de soins primaires. Cinq événements clés ont été retenus: (i) un moment charnière; (ii) la réalisation d'une synthèse des preuves scientifiques et la formulation de recommandations; (iii) la présentation de ces recommandations au comité responsable de la liste nationale de médicaments essentiels; (iv) l'intégration des recommandations en matière de réadaptation dans les rubriques correspondantes des directives thérapeutiques; et enfin, (v) la soumission des recommandations révisées au comité pour examen final. Le principal enseignement tiré est qu'en collaborant, les professionnels de la réadaptation peuvent être suffisamment nombreux pour faire la différence, améliorer la qualité des services et augmenter les transferts depuis les soins primaires vers les structures de réadaptation. Un défi subsiste: l'absence de représentants du secteur au sein du comité responsable de la liste nationale de médicaments essentiels, ce qui pourrait nuire à la compréhension de la réadaptation et du degré de complexité des faits qui l’étayent.

## Resumen

La Organización Mundial de la Salud reconoce que la rehabilitación es un componente esencial de la cobertura sanitaria universal (CSU). En muchos países, la cobertura sanitaria universal depende de un paquete estándar de servicios que se basa en la lista de medicamentos esenciales del país, las directrices de tratamiento estándar y la lista de laboratorios esenciales de atención primaria. En Sudáfrica, la atención primaria la prestan y gestionan en gran medida el personal de enfermería y los médicos de atención primaria, de conformidad con las directrices de tratamiento estándar de la atención primaria. Sin embargo, la rehabilitación queda excluida en su mayor parte de estas directrices. Este artículo describe el proceso de 10 años que llevó a que se contemplara la posibilidad de incluir las recomendaciones de derivación a rehabilitación en las directrices de tratamiento estándar de la atención primaria en Sudáfrica. Hubo cinco acontecimientos clave: (i) un momento decisivo; (ii) la elaboración de una síntesis de datos científicos y la formulación de recomendaciones; (iii) la presentación de las recomendaciones al comité de la lista nacional de medicamentos esenciales; (iv) la adaptación de las recomendaciones de rehabilitación a las secciones pertinentes de las directrices de tratamiento; y (v) la presentación de las recomendaciones revisadas al comité para su estudio final. La principal lección aprendida es que, trabajando juntos, los profesionales de la rehabilitación pueden ser lo bastante numerosos como para marcar la diferencia, mejorar la prestación de servicios y aumentar las derivaciones a la rehabilitación desde la atención primaria. Un desafío que aún persiste es que no hay un representante de la rehabilitación en el comité de la lista nacional de medicamentos esenciales, lo que podría dificultar la comprensión de la rehabilitación y de las complejidades de las pruebas que la sustentan.

## ملخص

تدرك منظمة الصحة العالمية أن إعادة التأهيل تعد مكونًا أساسيًا للتغطية الصحية الشاملة (UHC). ترتكز التغطية الصحية الشاملة في العديد من الدول على حزمة ذات مزايا قياسية من الخدمات، والتي تكون على دراية بقائمة الأدوية الأساسية للدولة، وإرشادات العلاج القياسية، وقائمة المختبرات الأساسية للرعاية الصحية الأولية. يتم في جنوب إفريقيا تقديم الرعاية الصحية الأولية وإدارتها إلى حد كبير بواسطة ممرضات الرعاية الصحية الأولية، والموظفين الطبيين، وفقًا لإرشادات العلاج القياسية للرعاية الصحية الأولية. ومع ذلك، فإن إعادة التأهيل يتم في الغالب استبعادها من هذه المبادئ الإرشادات. تصف هذه الورقة العملية التي استمرت لمدة 10 سنوات، والتي أدت إلى النظر في توصيات الإحالة لإعادة التأهيل بهدف تضمينها في إرشادات العلاج القياسي للرعاية الصحية الأولية في جنوب إفريقيا. كانت هناك خمسة أحداث رئيسية: (1) لحظة انفراج؛ و(2) إنتاج تجميع للأدلة العلمية وصياغة التوصيات؛ و(3) تقديم التوصيات إلى لجنة قائمة الأدوية الأساسية الوطنية؛ و(4) تطبيق توصيات إعادة التأهيل على أقسام إرشادات العلاج ذات الصلة؛ و(5) تقديم توصيات منقحة إلى اللجنة للنظر فيها بشكل نهائي. الدرس الرئيسي المستفاد هو أنه من خلال العمل معًا، يمكن أن يكون هناك عدد كافٍ من إخصائيي إعادة التأهيل لإحداث الفرق، وتحسين تقديم الخدمة، وزيادة الإحالات إلى إعادة التأهيل من الرعاية الصحية الأولية. التحدي الباقي هو الافتقار إلى وجود ممثل لإعادة التأهيل في لجنة قائمة الأدوية الأساسية الوطنية، مما قد يعيق فهم إعادة التأهيل وتعقيدات الأدلة الداعمة.

## 摘要

世卫组织认为康复治疗是全民健康覆盖 (UHC) 的重要组成部分。在许多国家，UHC 通过提供一揽子标准福利服务实现，这些服务项目根据国家基本药物清单、标准治疗指南和初级医疗保健服务基本实验室清单制定。在南非，初级卫生保健服务主要由初级卫生保健护士和医务人员根据初级卫生保健标准治疗指南提供和管理。然而，这些指南中大多不包含康复治疗。本文描述了历经 10 年将康复转诊建议纳入南非初级卫生保健标准治疗指南的过程。包含五个主要事件：(i) 突破性时刻；(ii) 提供综合科学证据并提出建议；(iii) 向国家基本药物目录工作委员会提出建议；(iv) 将康复治疗建议纳入相关治疗指南部分；以及 (v) 向委员会提交修改后的建议以供最终审议。可借鉴的主要经验是，康复治疗师可以通过合作弥补康复人才不足的问题、提高服务水平以及增加初级卫生保健的康复治疗转诊率。但问题是，国家基本药物目录工作委员会中缺少康复治疗师代表，这可能会妨碍理解康复治疗以及复杂的支持证据。

## Резюме

Всемирная организация здравоохранения признает реабилитацию важнейшим компонентом всеобщего охвата услугами здравоохранения (ВОУЗ). Во многих странах ВОУЗ основывается на стандартном пакете услуг, который основан на перечне основных лекарственных средств страны, стандартных руководящих принципах по лечению и перечне основных лабораторий первичной медико-санитарной помощи. В Южной Африке первичная медико-санитарная помощь в основном предоставляется и управляется медсестрами и медицинскими работниками служб первичной медико-санитарной помощи в соответствии со стандартными руководящими принципами лечения в рамках первичной медико-санитарной помощи. Однако реабилитация в основном исключена из этих руководящих принципов. В этом документе описывается 10-летний процесс, результатом которого стало рассмотрение рекомендаций по направлениям на реабилитацию для их включения в стандартные руководящие принципы лечения в рамках первичной медико-санитарной помощи в Южной Африке. Основных событий было пять: (i) момент прорыва; (ii) подготовка синтеза научных данных и формулирование рекомендаций; (iii) представление рекомендаций комитету по национальному перечню основных лекарственных средств; (iv) сопоставление рекомендаций по реабилитации с соответствующими разделами руководящих принципов по лечению; (v) передача пересмотренных рекомендаций комитету для окончательного рассмотрения. Главный вывод заключается в том, что специалистов по реабилитации нужно подготовить в достаточном количестве, чтобы совместными усилиями изменить ситуацию, улучшить предоставление услуг и увеличить количество направлений на реабилитацию из учреждений первичной медико-санитарной помощи. Нерешенной задачей является отсутствие представителя по реабилитации в комитете по национальному перечню основных лекарственных средств, что может затруднить понимание реабилитации и сложностей подтверждающих доказательств.

## Introduction

In 2017, the World Health Organization (WHO) issued a call for action on rehabilitation that urged coordinated, concerted global efforts to strengthen the place of rehabilitation in health systems.^1^ The barriers faced by people who need rehabilitation, particularly in low- and middle-income countries, are well-documented.^2^ They include high out-of-pocket transport costs, poorly coordinated or inappropriate health care, and health-care expenses that far exceed those of the general population.^3^^,^^4^ Moreover, WHO recognizes rehabilitation as an essential aspect of health care to be included in universal health coverage (UHC).^5^ Inclusion will ensure people receive care when and where they need it, without exposing them to financial hardship.^6^

UHC builds on a country’s so-called standard benefits package of services. In South Africa, as in many countries, this care package is based on the country’s essential medicines list, standard treatment guidelines, primary health care essential laboratory list and various national clinical protocols.^7^^,^^8^ In accordance with the evidence-based principles underlying WHO’s essential medicines list, South Africa’s standard treatment guidelines and essential medicines list aim to satisfy the needs of the population and guide physicians in prescribing rational, evidence-based treatment.^9^ Collectively, these guidelines, lists and protocols frame the comprehensive care provided to South Africans who depend on the public health sector. However, the country still lacks an explicit prioritization process and has, in the past, been heavily influenced by mortality profiles and donor agencies.

South Africa is classified as an upper-middle-income country but is marred by gross wealth inequality, high unemployment and low literacy. Health care is provided by an inefficient, chronically underfunded, public health-care system and a well-resourced, private health-care system. About 80% of the population depend on the public sector, which received only around 33% of all health-care spending in 2017 to 2018.^10^ Although the public system offers free care to children and elderly people, the quality is suboptimal and access is limited by an inadequate workforce and by the geographical inaccessibility of facilities, which provide the majority of care. The disparity in resources between public and private sectors has led to widespread inequity in the system, which means that people in the most vulnerable and poorest communities who have disabilities or who need help with functioning are widely neglected.

In South Africa, primary care is largely provided and managed by primary health-care nurses and medical officers. Although rehabilitation professionals, such as physiotherapists, may be an individual’s first contact with health care in the private sector, this does not occur in the public sector, which further deepens inequities. In the public sector, patients are only able to access rehabilitation by referral from a primary health-care nurse or medical officer. However, nurses and medical officers are generally overloaded and spend only around seven minutes with each patient.^11^ Moreover, even where rehabilitation services are available, high workloads and a lack of knowledge about rehabilitation may result in low referral rates. Consequently, the true need for rehabilitation has been underestimated in many communities.

Several publications have reported that the primary health care rehabilitation service and workforce are inadequate in South Africa.^12^^–^^16^ For example, the estimated number of occupational therapists in the country is less than one for every 10 000 people who depend on public health care.^17^ Furthermore, therapists are mainly available only in tertiary hospitals and less than a quarter of primary health-care facilities provide rehabilitation on a daily basis.^18^^,^^19^ In most provinces (especially rural provinces), rehabilitation services are provided through outreach programmes conducted at clinics, which often have limited space and infrastructure for rehabilitation. Primary health-care services in South Africa are frequently provided by relatively inexperienced, new graduates whose training requires them to work for a minimum of one year in the public sector before registering as an independent practitioner.^20^ Consequently, patients have reported that rehabilitation care is inadequate and ineffective due to poor organization and the limited competence of the workforce.^16^ In addition, the lack of standardized, evidence-based guidelines for primary health care has further reduced the likelihood of receiving good-quality rehabilitation services.^21^^,^^22^

## Rehabilitation in South Africa

The first democratic elections in South Africa, in 1994, marked the start of an era of hope for a better life for all, especially for poor and vulnerable groups. The new, democratic leadership spearheaded revisions to existing health service policies and commissioned new policies to improve access, particularly for marginalized communities. In 2000, the health minister developed the National Rehabilitation Policy (Box 1).^23^

Box 1 **Main objectives of the 2000 National Rehabilitation Policy, South Africa^23^**

- To improve the accessibility of rehabilitation services
- To establish mechanisms for intersectoral collaboration to implement a comprehensive rehabilitation programme
- To facilitate the appropriate allocation of resources and encourage their optimal utilization
- To facilitate human resource development that considers the needs of both service providers and consumers
- To encourage the development and implementation of monitoring and evaluation strategies for rehabilitation programmes
- To ensure participation of persons with disabilities in the planning, implementation and monitoring of rehabilitation programmes
- To encourage research initiatives in rehabilitation and related areas

**Fig. 1:**
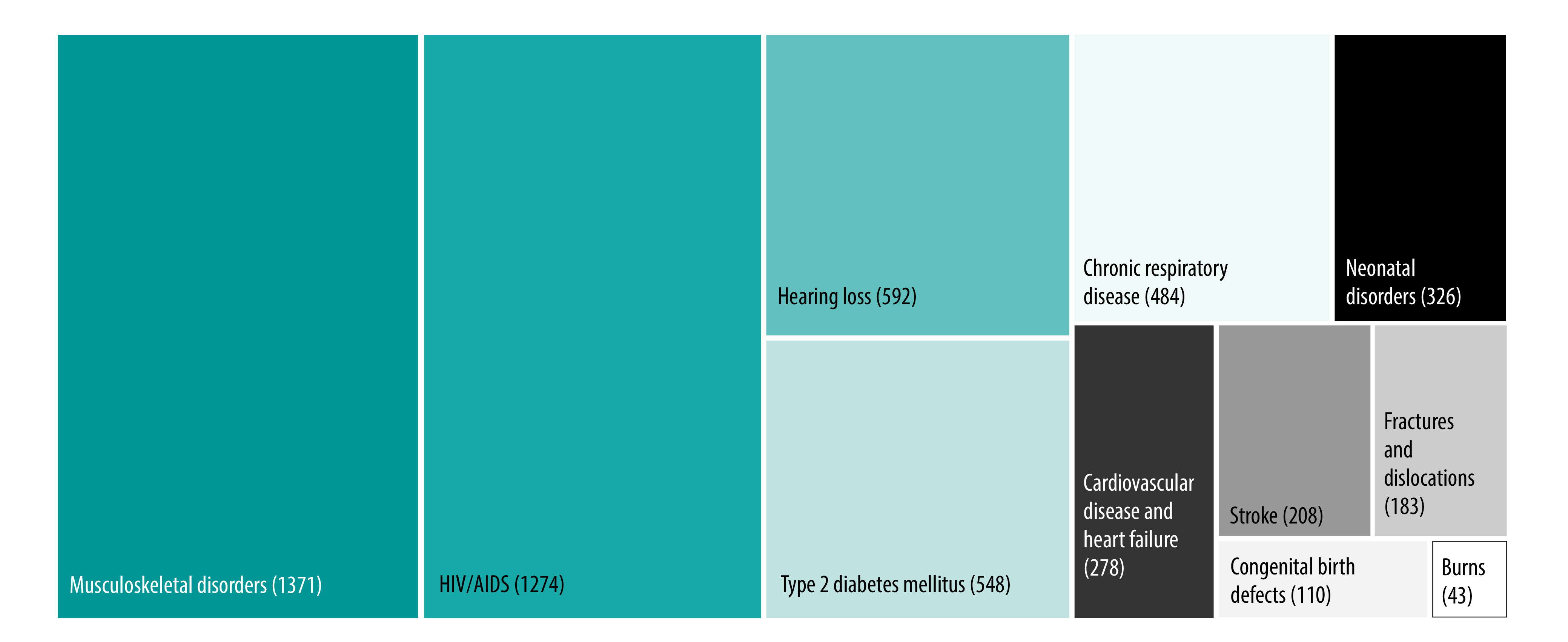
Years lived with disability, by clinical condition amenable to rehabilitation, South Africa, 2019

AIDS: acquired immunodeficiency syndrome; HIV: human immunodeficiency virus.

Note: The figures in parentheses represent the number of years lived with disability per 100 000 population based on 2019 Global Burden of Disease data for South Africa.^24^

South Africa has a unique epidemiological profile and the need for rehabilitation services is substantial.^24^ Although life expectancy increased between 1990 and 2017, the country is experiencing colliding epidemics of communicable and noncommunicable diseases, which have changed disease profiles and trends. In addition, people are living with increased morbidity and a poorer quality of life, both of which can be ameliorated by rehabilitation. A 2021 analysis found that almost 60% of all years lived with disability in South Africa were due to conditions amenable to rehabilitation, including (Fig. 1):^24^ (i) human immunodeficiency virus infection and acquired immunodeficiency syndrome (with resultant tuberculosis); (ii) chronic respiratory disease; (iii) type 2 diabetes mellitus; (iv) low back pain and other musculoskeletal disorders; (v) age-related and other hearing loss; (vi) neonatal disorders; (vii) congenital birth defects; (viii) lower limb, upper limb, spinal and multiple fractures and dislocations; (ix) cardiovascular disease and stroke; and (x) burns.

**Fig. 2:**
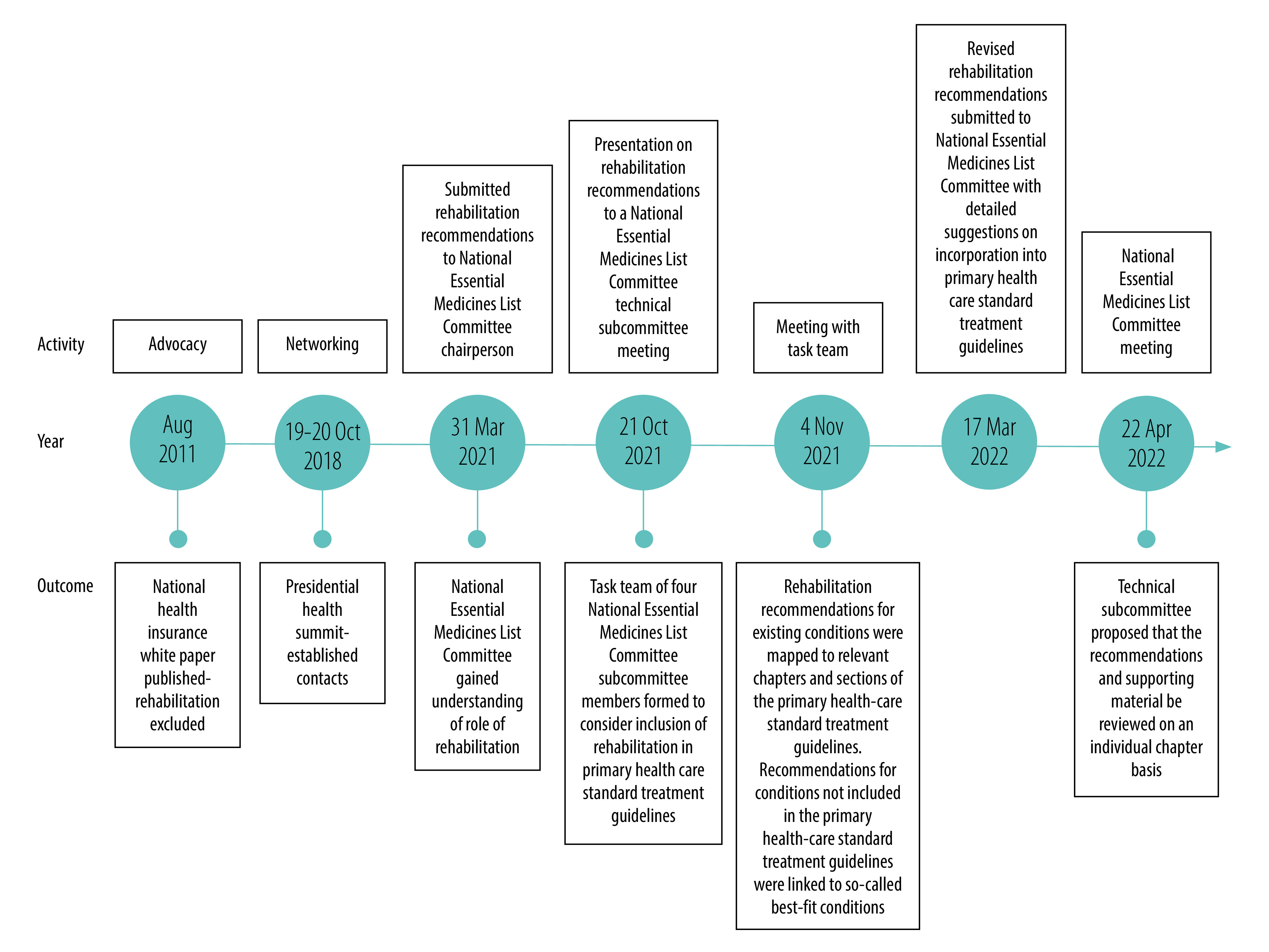
Actions by rehabilitation advocates and their outcomes, integration of rehabilitation into primary health care standard treatment guidelines, South Africa, 2011–2022

As primary health care has been identified as the vehicle for delivering health services to South Africans using the public sector, in 2017 the government initiated major health systems reforms to address the fragmented, inefficient and inequitable provision of health care in the country, particularly at the primary level.^25^ Traditionally, rehabilitation services have been incorporated into the health system in vertical programmes and there has been limited scope for integration into the priority health programmes that benefit from political support and increased resources, such as noncommunicable disease programmes.^26^ As South Africa began the transition to integrated care, one key goal of the Framework and Strategy for Disability and Rehabilitation Services in South Africa 2015 was to integrate comprehensive disability and rehabilitation services into priority health programmes from primary to tertiary and specialized health-care levels.^27^ The Framework and Strategy included practical strategies for operationalizing the national rehabilitation policy and provided a blueprint for promoting equitable and inclusive rehabilitation services through restructuring primary health care (Fig. 2).

In primary health care in South Africa, the care provided by nurses and medical officers is guided by standard treatment guidelines. However, rehabilitation is mostly excluded from these guidelines, though there are brief mentions. The resulting lack of direction on how to operationalize rehabilitation services within primary health care has had a negative impact on the capacity to deliver rehabilitation services and, consequently, on patients’ ability to access rehabilitation through primary health care. As South Africa moves towards a national health insurance system, there is a real risk that rehabilitation services will be insufficiently included and that people requiring rehabilitation will be unable to access appropriate care. Rehabilitation is underdeveloped at all levels of care in South Africa and the situation is dire at the primary care level.^28^ A crucial first step towards achieving UHC for people requiring rehabilitation is to integrate rehabilitation into the country’s standard treatment guidelines for primary health care, as advocated in the Framework and Strategy for Disability and Rehabilitation Services. The actions we describe in this paper provide an example of how existing national guidance can be leveraged to address this specific barrier to attaining the government’s stated health-care goal to leave no one behind.^29^^,^^30^

## Trigger for change

In August 2011, a government white paper on national health insurance was circulated for comment. Professional organizations, health policy-makers, clinical managers, clinicians, patients and researchers were all consulted. In addition, Rural Rehab South Africa and other professional bodies were involved in hearing the voices of health-care users and advocates – Rural Rehab South Africa is a multidisciplinary organization with a focus on health inequities and strong links with human rights organizations.

The glaring exclusion of rehabilitation from the white paper galvanized rehabilitation stakeholders into action. National rehabilitation bodies (Table 1) provided robust feedback to government officials and urged the inclusion of rehabilitation. Subsequently, these bodies consistently provided feedback on all policies and guidelines circulated for comment. One important consequence was a recognition that Rural Rehab South Africa should be consulted on future health policies. Although feedback and comments from Rural Rehab South Africa have yet to be considered for inclusion in national policies or guidelines, the consultations enabled the organization to develop relationships with key government officials. Additionally, the collective feedback from rehabilitation bodies has progressively heightened awareness about the exclusion of rehabilitation from national health policies and guidelines.

**Table 1:** Organizations involved in advocating for the integration of rehabilitation into primary health care standard treatment guidelines, South Africa, 2011–2022

Organization	Aim or mission	Vision
Occupational Therapy Association of South Africa^31^	The association aims to promote and represent the profession of occupational therapy as a key component of the health-care sector in South Africa	Occupational therapy as an integral, evidence-informed and relevant force meeting society’s occupational needs in partnership with key stakeholders and the public
Rural Rehab South Africa^32^	A multidisciplinary organization of professionals committed to providing and improving rehabilitation services in rural communities	To ensure that high-quality, comprehensive, appropriate, accessible and equitable rehabilitation services are provided within a primary health-care framework to all rural communities
South African Association of Audiologists^33^	In its quest to promote audiology and in striving towards excellence in professional service, the association actively liaises with related professional associations and bodies, statutory institutions (e.g. the Health Professions Council of South Africa) and health insurance companies, among others	To represent the eyes, ears, voice and conscience of the profession of audiology
South African Speech–Language–Hearing Association^34^	Promotes the professions to the public and promotes the interests of members in all spheres of professional activity. Lobbies and advocates to ensure recognition of the profession by government, the private sector, international bodies and health insurance schemes	To be the acknowledged voice of speech and language therapists and audiologists in South Africa
South African Society of Physiotherapy^35^	The society is a voluntary professional organization committed to equal opportunities and inclusivity that strives to reflect the demographic profile of South Africa in terms of race and gender	To be a dynamic, professional, innovative organization that represents its members effectively and plays a proactive role in the formulation and implementation of health-care policy in South Africa
Medical Orthotics and Prosthetics Association of South Africa (MOPASA)	NA	NA

NA: not available.

Between 2011 and 2022, rehabilitation advocates (Table 1 lists the main organizations) took part in several actions to ensure the integration of rehabilitation into primary health care standard treatment guidelines (Fig. 2). These are some of the key actions.

### Action 1

December 2020 marked a breakthrough moment when Trudy Leong from the South African National Department of Health’s Essential Drugs Programme requested Rural Rehab South Africa (on behalf of rehabilitation bodies) to submit recommendations on the inclusion of rehabilitation in primary health care standard treatment guidelines for consideration by the national essential medicines list committee. This committee is a non-statutory advisory committee appointed by government ministers that is responsible for developing and managing the national essential medicines list and standard treatment guidelines.^36^ In formulating its recommendations, Rural Rehab South Africa was assisted by professional rehabilitation bodies, including the Occupational Therapy Association of South Africa, the South African Speech–Language–Hearing Association, the Medical Orthotics and Prosthetics Association of South Africa, the South African Society of Physiotherapy and the South African Association of Audiologists (Table 1).

### Action 2

**Fig. 3:**
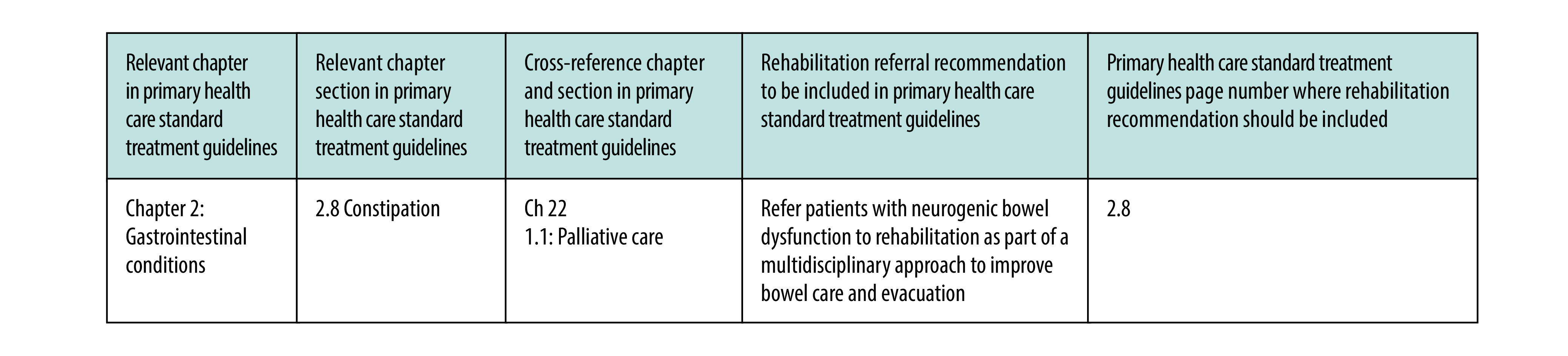
Example of rehabilitation referral recommendation in primary health care standard treatment guidelines, South Africa, 2022

**Fig. 4:**
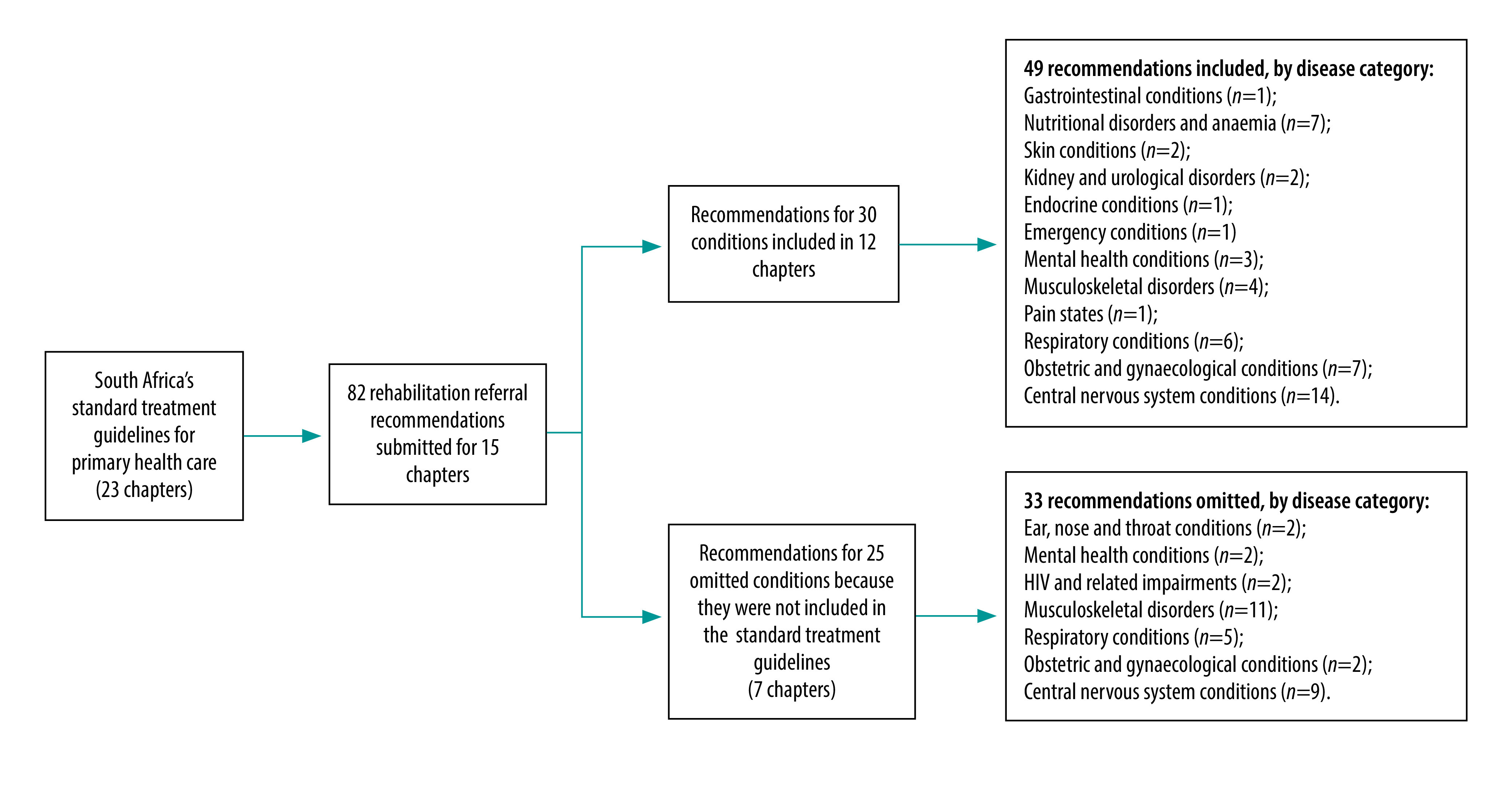
Rehabilitation referral recommendations submitted for inclusion in primary health care standard treatment guidelines, South Africa, 2021

In 2021, a research team based at Stellenbosch University and led by the South African Research Chair in Innovative Rehabilitation, Quinette Louw, steered a synthesis of the scientific evidence supporting the inclusion of rehabilitation into standard treatment guidelines for primary health care and formulated recommendations. The key questions on which evidence was sought were identified using the PICO (population, intervention, comparison and outcomes) method by Rural Rehab South Africa, leading clinicians and professional associations,^37^ who also developed the overarching principles of the research. There was a collective agreement that rehabilitation referral recommendations should be formulated for inclusion in standard treatment guidelines (Fig. 3 shows an example). These recommendations are critical for addressing the low rehabilitation referral rates in primary health care because the guidelines are mainly used by primary health-care nurses and medical officers.^8^ The Stellenbosch University team conducted a rapid review of the evidence between February and March 2021 and formulated 82 rehabilitation referral recommendations (underpinned by evidence of effectiveness) based on national standard essential drug programme and essential medicines list templates (Fig. 4).

HIV: human immunodeficiency virus.

Note: Some conditions were not included in the primary health care standard treatment guidelines but were covered by other South African standard treatment guidelines (e.g. adult standard treatment guidelines).

The Stellenbosch University team deemed it necessary to reflect on the challenges of developing evidence-based recommendations on rehabilitation for a national essential medicines list committee that consisted primarily of medical professionals. To ensure the committee had sufficient background information, we felt it was important to explain that rehabilitation was a complex process that involves a series of coordinated actions and multidisciplinary care provided using a patient-centred approach. Our intentions were supported by the increasing number of reviews on rehabilitation topics being published around the world, which reflected a global focus on the burden of disease posed by chronic conditions and demonstrated the keen interest in developing better understanding of effective rehabilitation.^38^^,^^39^ Therefore, we included a preface in our submission to the national essential medicines list committee that summarized advances in the method for generating, interpreting and grading evidence in rehabilitation. We highlighted key differences between rehabilitation research and medical or drug trials to justify our inclusion of recommendations underpinned by a lower level of evidence than would typically be used to support medical interventions.

### Action 3

In October 2021, Quinette Louw was invited by Trudy Leong to give a presentation to the technical subcommittee of the national essential medicines list committee (at a virtual meeting) explaining the rehabilitation recommendations submitted for inclusion in standard treatment guidelines. Quinette Louw outlined the preface to the submission and explained how the rehabilitation referral recommendations had been formulated. As this was uncharted terrain for the subcommittee, a smaller task team comprising four subcommittee members was formed to draft a methodological process that the committee could use to guide the inclusion of rehabilitation in standard treatment guidelines.

### Action 4

The technical subcommittee’s task team communicated by email and met online to discuss the most feasible strategy and method that the committee could use to decide on how and where to integrate rehabilitation recommendations into standard treatment guidelines. The primary health care standard treatment guidelines form a very large document of close to 1000 pages that are organized into chapters (by anatomical disorder group) and subsections. The team collectively developed a strategy for mapping rehabilitation recommendations onto the relevant guideline chapters or sections and a date was set to ensure that the mapping strategy was considered at the next technical subcommittee meeting.

### Action 5

In March 2022, the Stellenbosch University research team, in collaboration with Rural Rehab South Africa, submitted a revised document (281 pages) that clearly outlined the rehabilitation recommendations and where they should be incorporated into primary health care standard treatment guidelines. The document included a table with hyperlinks to the evidence and associated references supporting each recommendation (cross-references to relevant chapters for each recommendation were also indicated). The technical subcommittee considered the revised rehabilitation referral recommendations in April 2022 and proposed that the recommendations and supporting material be reviewed on an individual chapter basis (according to a prioritization plan) before submission to the national essential medicines list committee for further review and ratification. At the time of writing, nephrological conditions had been reviewed. Note that the review process for the national essential medicines list is iterative and that amendments are circulated for external peer review before being adopted and finalized for publication.

## Discussion

**Fig. 5:**
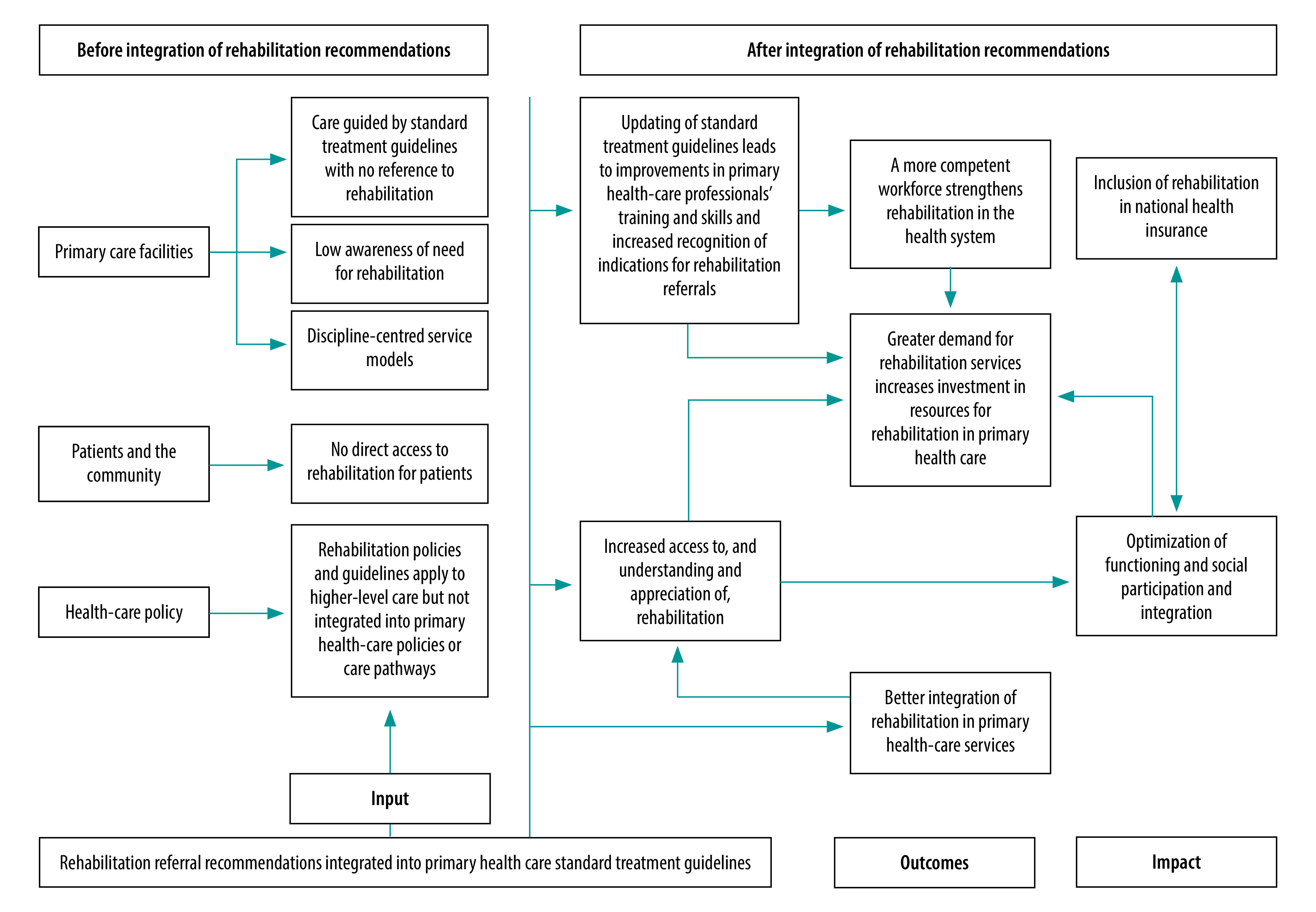
Expected effect on health system of integrating rehabilitation recommendations into primary health care standard treatment guidelines, South Africa, 2022

This is the first time evidence-based rehabilitation recommendations will be included in standard treatment guidelines in South Africa, thereby cementing the legal right to rehabilitation services through primary care and positively influencing people’s functioning and participation in society. Fig. 5 illustrates how the integration of rehabilitation referral recommendations into standard treatment guidelines for primary health care tackled existing shortcomings and improved outcomes. Ultimately, the integration of rehabilitation into these guidelines, which anchor the health-care services provided in the public sector, should lead to coverage of rehabilitation by the forthcoming national health insurance, which will help ensure that the public health system better serves the rehabilitation needs of South Africans. 

During the consultation process, the national essential medicines list committee gained a broader insight into rehabilitation and greater understanding of the supporting evidence. Subsequently, the committee proposed that the process followed for rehabilitation be used as a blueprint for the inclusion of other health strategies (e.g. palliation) in guidelines. This unplanned outcome was welcome.

This process through which rehabilitation recommendations were incorporated into standard treatment guidelines is a considerable accomplishment, which involved more than 10 years of raising awareness, advocacy, strategy formulation and engagement with policy-makers (Fig. 2). Ironically, it also marked the beginning of a new journey towards strengthening policy on, and the implementation of, rehabilitation. The process had some unique features: the right opportunities were seized at the right time and a multidisciplinary, national partnership made a coordinated, collective effort to achieve a common goal.

All members of the partnership played crucial roles and the success of the process was due to the united actions of a diverse, critical mass of health professionals with the patience and determination to bring about change. Another vital factor was leadership by self-motivated professionals with experience in implementing health-care policies and the ability to deliver outcomes timeously when the opportunity arose.

### Lessons learnt

Several valuable lessons were learnt (Box 2). First, rehabilitation professionals should respond to invitations to comment on high-level policy documents. Reviewing and providing feedback on such documents provides insights into the policy frameworks that could potentially serve as avenues for strengthening rehabilitation. In addition, feedback from rehabilitation professionals increases the visibility of rehabilitation.

Box 2 **Main lessons learnt, integration of rehabilitation into primary health care standard treatment guidelines, South Africa, 2011–2022**

- Rehabilitation professionals should strive to contribute to high-level, national policy documents and highlight the absence of references to rehabilitation where appropriate
- Advocacy efforts must be timely and aligned with national strategic goals or directions
- United we stand, divided we fall – rehabilitation professionals should act collectively because acting only within their own areas of expertise may not produce the critical mass needed to be effective in strengthening rehabilitation in the health system
- Recommendations must be reinforced by sound scientific evidence to secure support from scientific bodies and decision-makers
- Although rehabilitation is patient-centred, rehabilitation professionals should intensify and sharpen their networking skills
- Effective advocacy depends on meticulous project management, meeting deadlines and responding when the opportunity arises
- Rehabilitation professionals should be included on national standard treatment guideline panels to strengthen rehabilitation in the health-care system
- A younger generation of health professionals should be nurtured to promote change, thereby ensuring that rehabilitation will be included in the health-care system in the future

Second, an integrated approach can be powerful. It can be ineffective for professionals in countries with weak health systems and constrained resources to act only within their own areas of expertise. Rehabilitation professionals should intensify and sharpen their networking skills to ensure rehabilitation becomes a priority health strategy. In South Africa, we may have wasted considerable time by failing to engage all relevant stakeholders, although our collective efforts did ultimately have an impact on the health system.

Third, advocacy must be timely and aligned with strategic, national goals if change is to be achieved. Moreover, although advocacy is important, health-care recommendations must be reinforced by sound scientific evidence to secure support from scientific bodies and decision-makers. Partnerships within and outside the health system are important for maximizing the impact of advocacy in a cost-effective manner. The effectiveness of a partnership also benefits from its members communicating effectively and having a diverse range of backgrounds and skills, as well as shared values.

Our experience demonstrates that considerable work is needed to strengthen rehabilitation in primary health care as every step we took revealed further gaps in the health-care system in South Africa, which explains why rehabilitation services in the country remain suboptimal. We acknowledge that improving access to, and the quality of, rehabilitation in primary health care involves a complex web of multipronged strategies that must be implemented over time. Thus, a younger generation of health professionals should be nurtured to promote change and ensure that rehabilitation will continue to be included in the health-care system in the future.

One remaining challenge is the lack of a rehabilitation representative on the national essential medicines list committee, which is instrumental in making national decisions on standards and care packages in South Africa. However, the presentation on rehabilitation Quinette Louw gave to the committee and the preface to the submission on rehabilitation referral recommendations increased the committee’s understanding of rehabilitation issues and of the complexities of the supporting evidence. Nevertheless, we will continue to advocate the inclusion of a rehabilitation representative on the committee. 

Although the national essential medicines list committee asked for rehabilitation recommendations to be mapped onto primary health care standard treatment guidelines, the updated guidelines omitted recommendations that had been formulated for 25 conditions (based on priorities identified by clinicians and associations). In response, it was decided to link these 25 recommendations to so-called best-fit conditions but these omissions will have to be addressed in the future.

## Conclusions

Strengthening rehabilitation and its integration into national treatment guidelines are challenges not only for South Africa but for every country around the world. The process described in this paper marks a pivotal moment for our country that should result in tangible improvements to rehabilitation in primary health care. Although many changes were needed, we were forced to prioritize the inclusion of rehabilitation referral recommendations in standard treatment guidelines, which we believe will generate further opportunities. All stakeholders involved in the process will be informed about its outcome and we recommend that this information also be distributed to all relevant health facilities. In addition, primary health-care professionals will be encouraged (e.g. through social media) to engage with the standard treatment guidelines. We also plan continuous professional training for primary health-care professionals. Further advocacy will be required to ensure primary health care standard treatment guidelines include the rehabilitation recommendations currently omitted for some conditions. The next step involves providing specific rehabilitation recommendations (e.g. a WHO primary health-care package)^40^ alongside referral recommendations. We hope the case study presented here offers lessons and provides guidance to other countries endeavouring to incorporate rehabilitation into national policies and guidelines.
